# Automatic Detection and Classification of Epileptic Seizures from EEG Data: Finding Optimal Acquisition Settings and Testing Interpretable Machine Learning Approach

**DOI:** 10.3390/biomedicines11092370

**Published:** 2023-08-24

**Authors:** Yauhen Statsenko, Vladimir Babushkin, Tatsiana Talako, Tetiana Kurbatova, Darya Smetanina, Gillian Lylian Simiyu, Tetiana Habuza, Fatima Ismail, Taleb M. Almansoori, Klaus N.-V. Gorkom, Miklós Szólics, Ali Hassan, Milos Ljubisavljevic

**Affiliations:** 1 Radiology Department, College of Medicine and Health Sciences, United Arab Emirates University, Al Ain P.O. Box 15551, United Arab Emirates; 2 Medical Imaging Platform, ASPIRE Precision Medicine Research Institute Abu Dhabi, Al Ain P.O. Box 15551, United Arab Emirates; 3 Big Data Analytics Center, United Arab Emirates University, Al Ain P.O. Box 15551, United Arab Emirates; 4 Department of Oncohematology, Minsk Scientific and Practical Center for Surgery, Transplantology and Hematology, 220089 Minsk, Belarus; 5 Department of Computer Science and Software Engineering, College of Information Technology, United Arab Emirates University, Al Ain P.O. Box 15551, United Arab Emirates; 6 Pediatric Department, College of Medicine and Health Sciences, United Arab Emirates University, Al Ain P.O. Box 15551, United Arab Emirates; 7 Neurology Division, Medicine Department, Tawam Hospital, Al Ain P.O. Box 15258, United Arab Emirates; 8 Internal Medicine Department, College of Medicine and Health Sciences, United Arab Emirates University, Al Ain P.O. Box 15551, United Arab Emirates; 9 Physiology Department, College of Medicine and Health Sciences, United Arab Emirates University, Al Ain P.O. Box 15551, United Arab Emirates; milos@uaeu.ac.ae; 10 Neuroscience Platform, ASPIRE Precision Medicine Research Institute Abu Dhabi, Al Ain P.O. Box 15551, United Arab Emirates

**Keywords:** deep learning, interpretable machine learning, activation maximization, epileptic seizure, EEG, acquisition settings, source reconstruction

## Abstract

Deep learning (DL) is emerging as a successful technique for automatic detection and differentiation of spontaneous seizures that may otherwise be missed or misclassified. Herein, we propose a system architecture based on top-performing DL models for binary and multigroup classifications with the non-overlapping window technique, which we tested on the TUSZ dataset. The system accurately detects seizure episodes (87.7% Sn, 91.16% Sp) and carefully distinguishes eight seizure types (95–100% Acc). An increase in EEG sampling rate from 50 to 250 Hz boosted model performance: the precision of seizure detection rose by 5%, and seizure differentiation by 7%. A low sampling rate is a reasonable solution for training reliable models with EEG data. Decreasing the number of EEG electrodes from 21 to 8 did not affect seizure detection but worsened seizure differentiation significantly: 98.24 ± 0.17 vs. 85.14 ± 3.14% recall. In detecting epileptic episodes, all electrodes provided equally informative input, but in seizure differentiation, their informative value varied. We improved model explainability with interpretable ML. Activation maximization highlighted the presence of EEG patterns specific to eight seizure types. Cortical projection of epileptic sources depicted differences between generalized and focal seizures. Interpretable ML techniques confirmed that our system recognizes biologically meaningful features as indicators of epileptic activity in EEG.

## 1. Introduction

Epilepsy affects around 50 million people globally, making it one of the most common neurological diseases. The disability rate in epilepsy patients ranges from 0.2 to 5.8%. The risk of social maladjustment and stigmatization due to this disease is still high [1,2]. The main symptom of epilepsy is recurring, spontaneous seizures that are hard to detect and differentiate by type without an automatic AI-based solution.

The interpretation of electroencephalography (EEG) is challenging; therefore, around 25% of patients are misdiagnosed with epilepsy due to over-reading of benign EEG features [3,4]. This stimulates research on automatic seizure detection and differentiation systems. Automatic AI-based solutions may help to improve the sensitivity and specificity of the detection of electrophoretic events suggestive of epileptic seizures. However, the currently existing systems for EEG analysis suffer from the following setbacks. First, the spatial resolution of EEG is too low to accurately specify the location of an epileptogenic source and to discern between normal and abnormal neuronal activity of brain areas [5]. Second, data preprocessing depends heavily on artifact and noise removal [6]. Third, scalp EEG is dominantly used due to its non-invasiveness, although it is less sensitive than intracranial EEG [7]. Fourth, scalp epileptiform discharges do not accurately reflect the severity of epilepsy [8]. Fifth, EEG findings are hard to interpret and visualize because of the large amount of data [9]. Therefore, the identification of epileptic activity with traditional methods is time-consuming and operator-dependent. For these reasons, the need for an optimized algorithm for seizure detection is increasing. Such an algorithm applied to daily EEG monitoring with wearable devices can report to neurologists on the epileptic episodes that go unmentioned by the patient due to postictal amnesia. In the current study, we analyzed and tested approaches to optimize the automatic detection of epileptic activity and to differentiate among electrographic signatures of seizure subtypes.

Currently, machine learning (ML) and deep learning (DL) are used for automatic analysis of EEG. They recognize the diagnostic signs and patterns that otherwise remain unmentioned. However, the black-box nature of DL models offers no insight into exactly how decisions are made [10,11]. Typically, researchers fail to explain the models they receive, which serves as a barrier to the widespread clinical adoption of ML and DL models. Interpretable ML overcomes this limitation by providing an explanation for black box models or by visualizing diagnostic patterns. This strengthens the diagnostic and clinical value of ML and DL algorithms. With the interpretable approach, data scientists display EEG findings as heatmaps that demonstrate the contribution of input from each electrode to the final decision [12,13]. The data illustrated in the maps may evidence discernible EEG patterns in study cohorts, offering valuable new insights for future work [14]. Layers of interpretable neural networks can depict the weights learned from signal processing computations. In this case, they serve as spatial and frequency sub-band filters, and the extracted data are no longer abstract. Layer-wise relevance propagation reveals important features and explains the computations leading to decisions [15]. Despite the evident progress in image-based applications, building interpretable pattern classifiers for waveform data continues to be a challenging task. Furthermore, few researchers have developed interpretable DL neural networks applicable to epileptic seizure detection in EEG recordings [11].

*The aim of this study* is to find a set of approaches to improve the automatic detection and classification of epileptic seizures based on EEG data. In particular, we want to test optimal settings for acquiring and processing EEG data with ML algorithms. The targeted settings would provide researchers with accurate detection and classification of epileptic episodes into major types.

In order to achieve the specified goals, we accomplished the following tasks:

1. Select the optimal system architecture for seizure detection and classification, train the corresponding models, and test their performance on an open-source dataset;
2. Compare the accuracy of ML-based seizure detection and classification according to the EEG sampling frequency;
3. Determine if the accuracy of seizure detection and classification depends on the number of EEG electrodes;
4. Apply the activation maximization technique to interpretable ML for seizure detection and classification;
5. Differentiate among seizure types according to the location of electrophysiologic activity in the brain through the scalp.

## 2. Materials and Methods

### 2.1. Methodology

Working on the first part of objective one, we studied the existing techniques for automatic seizure detection and multigroup classification. To select an ML algorithm for our study, we applied the following criteria. First, the optimal model should be highly sensitive and specific after training on the scalp EEG data. Second, the model should be able to generalize on a broad set of EEG recordings. It should be able to detect and classify different types of seizures. Third, the model should determine seizures quickly to be implemented in real clinical settings. Finally, the model should identify easily interpretable patterns and dependencies that could be transparently applied in practice.

The second part of objective one was dedicated to system accuracy. To assess it, we trained models in the 5-fold cross-validation technique. For the binary classification models, we used the following performance metrics: sensitivity, specificity, precision, and F1 score. For the multigroup classification, we inspected accuracy (Acc), precision, recall and F1 score. We evaluated feature importance with mutual information (MI) metrics averaged over 100 runs. The dataset we used presented different seizure types disproportionally. Therefore, we calculated the weights that take into account the imbalanced distribution of the classes and passed the weights to the model at the training stage.

In the second objective, we determined how the sampling frequency affects model prediction. To achieve this goal, we ran the binary and multigroup classification model on a dataset in which the sampling rate (SR) of EEG recording was reduced from 250 to 50 Hz with a decrement of 50 Hz. To evaluate the quality of the binary and multigroup classifications, we used the same metrics as in the first objective.

**Figure 1:**
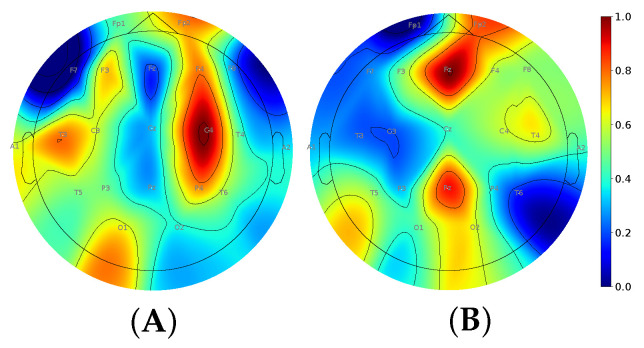
MI significance scores of EEG electrodes in binary (**A**) and multigroup classification (**B**).

In the third objective, we assessed the binary and multigroup classification models trained on the EEG data that were acquired with varying numbers of sensors. To calculate the importance of different EEG electrodes, we randomly selected 1000 windows of 21×250, calculated the MI scores and ranked the electrodes by their informative value. The MI values slightly varied in the binary and multigroup classifications: 0.0056–0.0068 and 0.172–0.178, respectively. Hence, the data coming from all EEG sensors were of almost equal informative value. We normalized MI scores and plotted them to the interval [0,1] (see Figure 1). For the binary classification model, the top informative electrodes were allocated paramedially at the left hemisphere (C4, F4, P4). For the multigroup classification, they were placed medially at the frontal and parietal cortex (Fz, Pz).

To define the minimal number of sensors required to detect seizures reliably, we first trained the binary classification model on the full 10/20 montage. Then we trained it on the reduced montage decreasing the number of electrodes one by one. At each step, we removed the electrode with the minimal MI value from the analysis. To assess the accuracy of the multigroup classification, we tested only two systems: with 8 and 21 electrodes correspondingly. Acting on a recommendation of Shah V. et al. [16], we selected F7, T3, C3, Cz, Fp2, F8, O2 and P4 electrodes out of the set of 21 sensors. The performance metrics were recorded at each fold of training, which resulted in a total number of 5 values per each metric. We assessed the difference between the model outcomes in 8- and 21-electrode montages with the parametric Welsh’s *t*-test and the non-parametric Mann–Whitney U test. D’Agostino’s and Pearson’s test was used to estimate the distribution of the metrics.

In the fourth objective, we used activation maximization (AM) as a method to visualize the features learned by neurons/filters in neural networks [17]. This approach enables researchers to highlight the data patterns that activate the neurons in the layer of interest. When applied to the last dense layer of the convolutional neural network (CNN), AM generates an image of the same dimensionality as the input. Each pixel of the generated image depicts the maximized activation in the output layer [18]. In our study, we applied AM to the last (dense) layer of the CNN and showed the patterns that help the network to detect seizure/non-seizure episodes and distinguish among different seizure types. We ran the AM algorithm with 100 iterations, and the resulting images were averaged over this number of runs.

In the fifth objective, we studied the brain electrophysiologic activity with a source reconstruction technique called Dynamic Statistical Parametric Mapping (DSPM).

### 2.2. Dataset Description

We used the Temple University Hospital EEG Seizure Corpus dataset (TUSZ) that contains information on 674 patients with epilepsy. The EEG data are stored as .edf files and medical reports are kept as .txt files. Generalized non-specific seizures (GNSZ), focal non-specific seizures (FNSZ), and complex partial seizures (CPSZ) are the most common epileptic episodes in the dataset. Other seizures are less presented in the TUSZ records: simple partial seizure (SPSZ), absence seizure (ABSZ), tonic seizure (TNSZ), tonic-clonic seizure (TCSZ), and myoclonic seizure (MYSZ). GNSZ, ABSZ, TNSZ, TCSZ and MYSZ are generalized seizures, while FNSZ, SPSZ and CPSZ are the focal ones. The SR and the number of electrodes differ among the records. For this reason, the records with a frequency over 250 Hz were downsampled. We analyzed 21 channels of the standard 10/20 montage.

### 2.3. Data Preprocessing

Data preprocessing did not differ between the seizure detection and seizure classification models. To determine the electrophysiologic activity characteristic of each seizure type, we focused on studying the episodes of high epileptiform activity that were more common in patients with multiple long-lasting seizures. Therefore, we selected 33 patients with the longest total duration of epileptic episodes (724 episodes overall). The raw EEG signals were processed with a 40 Hz finite impulse response filter and downsampled to 250 Hz SR. Then we used 21 electrodes of the standard 10/20 montage. We analyzed annotations in .tse files to identify time intervals of different seizures and episodes with normal brain activity. At the next step, the EEG data were cut into one-second-long sliding windows. The sequential sliding windows did not overlap, and we avoided data leakage. To increase the signal-to-noise ratio the raw records in the obtained windows were converted into the time-frequency domain with 64-point short-time Fourier transform. The resulting tensor with 21 channels, 8 timesteps and 33 frequencies was used as an input to each model. Finally, we divided the dataset into the training, testing and validation subsets.

### 2.4. CNN Model Architecture

Here we provide a concise description and diagram of the model architecture. Section S1 of the Supplemental Material contains a detailed explanation of the input/output data, methods and evaluation metrics used in the study.

#### 2.4.1. Binary Classification

**Figure 2:**
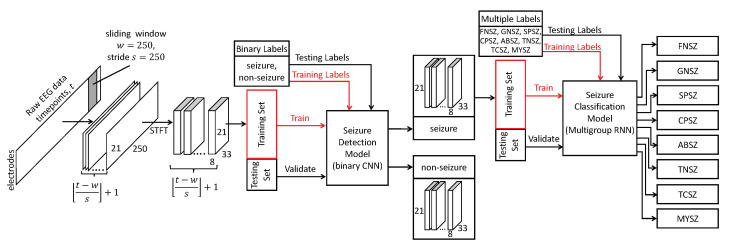
Architecture of system for seizure detection and classification.

The analysis of existing solutions for automatic seizure detection highlighted the strengths of a 3-layer 2D CNN model developed by M. H. Aslam et al. [19]. This binary classification model extracts distinguishing features of epileptiform activity in EEG data, and the model architecture allows us to implement the interpretable ML and visualize class patterns. The binary classification algorithm requires the same format of the input data as the multigroup classification model. This enables us to incorporate both models in a single pipeline, where the binary classification detects the seizure and the multigroup classification determines its type (see Figure 2).

The model consists of three 2D 3×3 convolutional layers of 16, 32 and 64 channels. The model output is a vector of activations the maximal value of which corresponds to the class of the probed region. The first convolutional layer with Rectified Linear Unit (ReLU) activation output is set to 16 channels, i.e., it extracts 16 feature maps. A 2D max pooling layer with 2×2 kernel follows the first convolutional layer. The second convolutional layer with ReLU activation and 32 channels is followed by another 2×2 max pooling layer. The third convolutional layer with ReLU activation outputs 64 channels. Next to this, there is another max pooling layer followed by flattening and two connected dense layers of 256 and 2 neurons with ReLU and Softmax activations correspondingly. We added the padding of 1 to both sides of the input to all convolutional layers. This provided us with the same spatial dimensions at the output, excluding channels. To accelerate the training process, the outputs from the max pooling layers were standardized with batch normalization. The accuracy, area under the receiver operating characteristic curve, recall, and precision metrics were used for model training in the Adam optimizer.

#### 2.4.2. Multigroup Seizure Classification

As a prototype of the multigroup classification model, we used a solution by T. Liu et al. [20] due to its high accuracy and relatively simple architecture. The latter contains two consecutive modules of a common structure: a convolutional long short-term memory (LSTM) layer that captures spatiotemporal correlations and preserves the input dimensions, a batch normalization layer, a max pooling layer, and a dropout layer. These modules are followed by a flattening layer and two dense layers of 256 and 8 neurons. While replicating the recurrent neural network (RNN) model for multiclass seizure detection, we resized the model input to make its dimensionality the same as in the binary classification. Unlike T. Liu et al., we used non-overlapping windows.

### 2.5. Source Reconstruction Technique

We used DSPM because it has advantages over other source localization techniques such as minimum norm estimation and low-resolution brain electromagnetic tomography (LORETA). DSPM can obtain spatiotemporal source distribution with a high temporal resolution [21]. Furthermore, the method can detect weak connections between active brain regions due to the application of noise covariance for normalization [22]. These facts make DSPM a promising tool for detecting epileptiform sources from EEG measurements. We projected the brain activity signals onto the surface of hemispheres of a T1 MRI template of an average subject from the MNE-Python library [23]. The library allowed us to estimate and visualize source time courses by calculating the forward solution and the inverse operator. For the baseline correction, we used the records obtained 100 ms before and 200 ms after the seizure. Since the idea of the study was to discriminate among seizure types by EEG data, we reconstructed source activity at the seizure onset and averaged the results over the first two seconds.

## 3. Results

### 3.1. Model Performance

**Figure 3:**
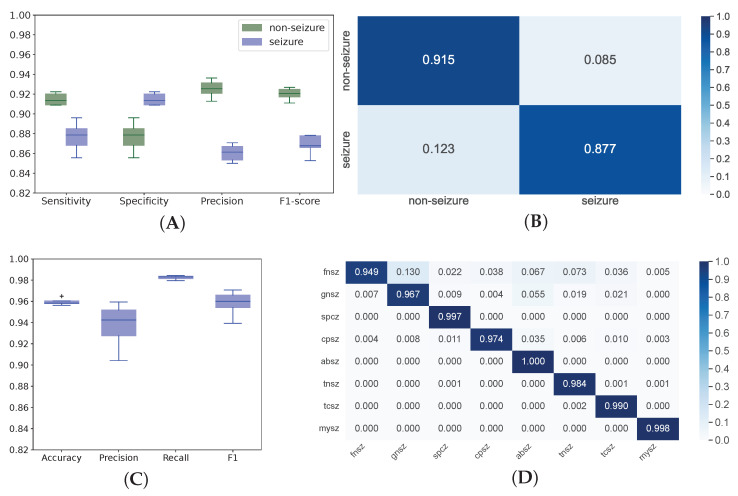
Performance of binary classification models (**A**)—performance metrics; (**B**)—normalized confusion matrix and multigroup classification models (**C**)—performance metrics averaged across all classes; (**D**)—normalized confusion matrix.

Analysis of relevant studies shows a marked trend towards applying DL instead of conventional ML to automatic seizure detection (see Table 1). DL models are easy to use since they accept raw data either without preprocessing or after minor corrections. Contrarily, most conventional methods require thorough data cleaning, artifact removal and additional feature engineering. DL networks are also easy to implement into practice, and they have reputable performance. As seen from the confusion matrix, training for 20 epochs was adequate to achieve good performance in the binary classification between *seizure* and *non-seizure* episodes: Sn 87.7% and Sp 91.16% (see Figure 3A,B). The multigroup classification model showed convincing performance after 50 epochs: Acc averaged across the classes was 96% (see Figure 3C,D). The model detected MYSZ most precisely, and it identified GNSZ with the least precision: 99% and 87% Acc, respectively. All ABSZ episodes were identified correctly while the lowest recall was detected in the FNSZ class (95%). The unequal distribution of cases in the training set accounts for the variance in precision and recall among the classes.

### 3.2. EEG Sampling Frequency in Seizure Detection and Classification

**Figure 4:**
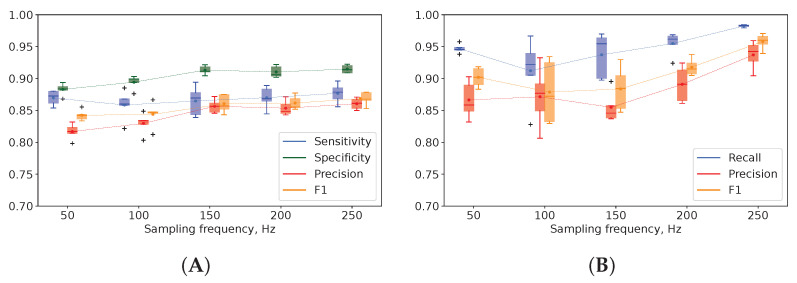
Evaluation of binary (**A**) and multigroup classification models (**B**) in terms of sensitivity, specificity, overall accuracy and positive class precision for different sampling rates.

We did not find a considerable variance in the performance of the binary and multigroup classification models trained on the data downsampled from 250 Hz to 50 Hz with a 50 Hz decrement (see Figure 4). However, the model accuracy slightly improved when we used a higher SR (250 Hz).

### 3.3. Number of EEG Electrodes in Automatic Detection and Classification of Epileptic Episodes

**Figure 5:**
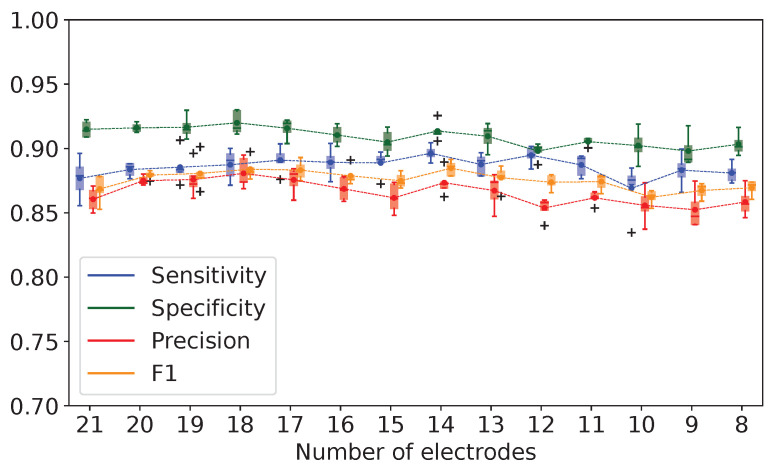
Performance of binary classification model with reduced number of electrodes.

We approached the problem of electrode selection in several ways. We calculated the informative value of the data coming from each electrode to distinguish between seizure and non-seizure activities and to categorize epileptic episodes into types (see Table 2). To start with, the binary classification model was trained on the EEG findings acquired with a varying number of sensors (from 21 to 8). In the experiment, we did not take into account the electrodes with minimal MI value (see Figure 5). It appeared that the binary classification model identified seizure episodes irrespective of the number of sensors.

Our approach was applicable for seizure detection, but we found it inapplicable for multigroup classification in which the sensors should cover the scalp to ensure accurate seizure categorization into classes. To cure the problem of dimensionality and find a minimal number of channels providing reasonable performance, we followed a suggestion of the researchers who optimized the selection of channels [16]. In their study, 8-channel systems had high specificity with overall performance close to a 16-channel system. These literature findings served as a major argument in favor of the comparison between 21- and 8-electrode systems in our study.

**Figure 6:**
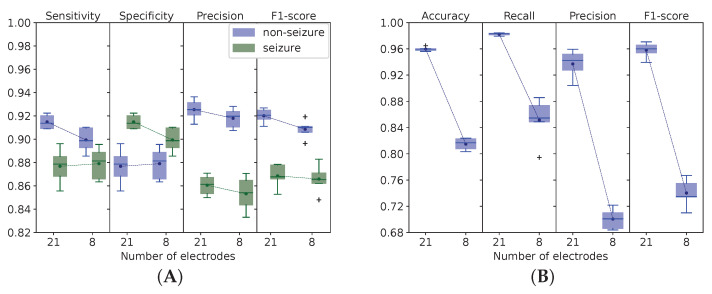
Performance of binary (**A**) and multigroup (**B**) classification models trained on EEG data received with 8 and 21 electrodes. Values on figure (**B**) averaged across 8 classes for each fold.

In the binary classification, we observed equal performance of the models trained on the EEG data acquired with different numbers of electrodes (see Figure 6A). When trained on the EEG data received with 21 electrodes, the classification algorithm had similar performance compared to the model trained on 8 electrodes: 87.69 vs. 87.90% Sn and 91.49 vs. 89.94% Sp. However, the quality of the RNN multigroup classification worsened with a decrease in the number of EEG detectors from 21 to 8 (see Figure 6B). Performance dropped considerably at the level of *p* < 0.001: 95.96±0.29 vs. 81.49±0.82% Acc; 93.71±1.96 vs. 70.05±1.43% precision; 98.24±0.17 vs. 85.14±3.14% recall; 0.95 ± 0.01 vs. 0.74 ± 0.02 F1 score.

### 3.4. Interpretable Machine Learning for Seizure Detection and Classification with Activation Maximization

**Figure 7:**
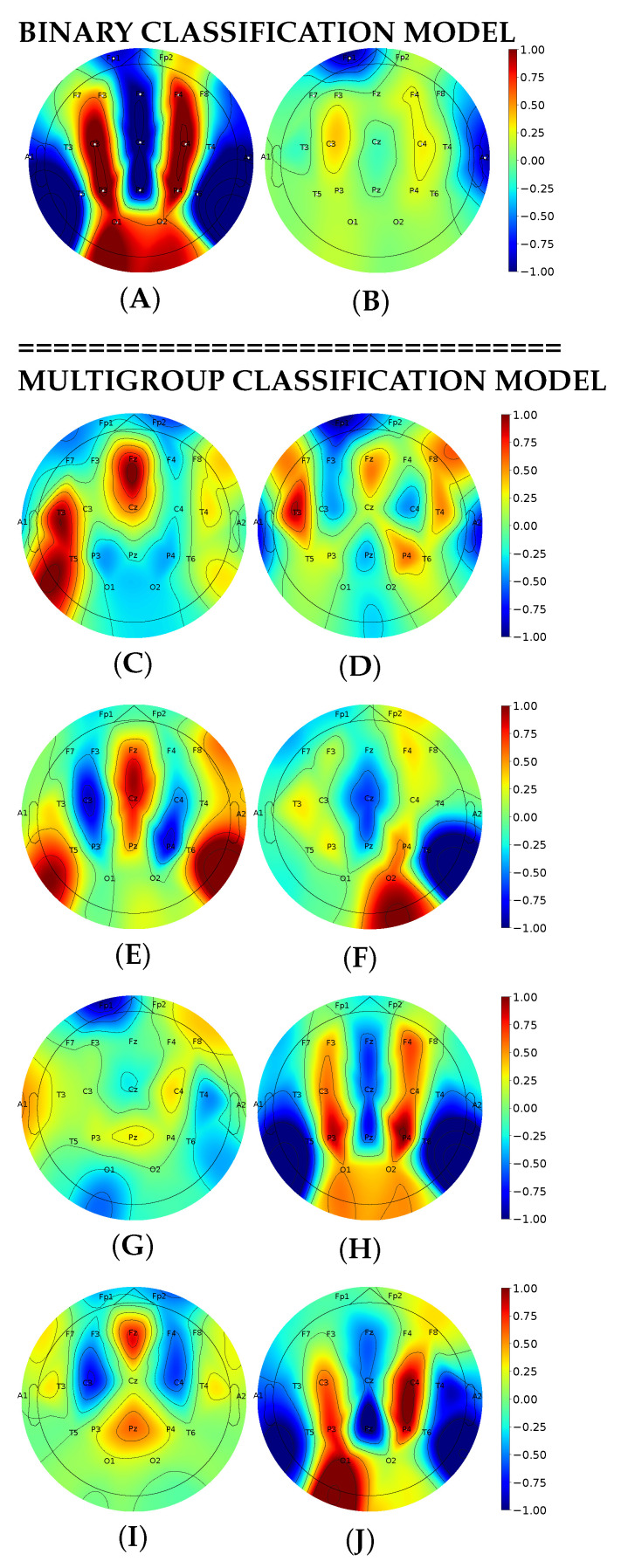
Visualization of model inputs with activation maximization in binary and multigroup classifications: seizure (**A**) and non-seizure episodes (**B**); FNSZ (**C**), SPSZ (**D**), CPSZ (**E**), MYSZ (**F**), GNSZ (**G**), ABSZ (**H**), TNSZ (**I**) and TCSZ (**J**).

The AM technique revealed the model inputs that were crucial for correct classification. With AM, we visualized the spatial filters that extracted the most important EEG features. The filters contained the information which helped the systems to differentiate between seizure and non-seizure states (see Figure 7A,B). They reduced background noise, and neural networks focused on the most important channels, thus increasing classification performance.

A possible interpretation of class-specific features for seizure/non-seizure detection is as follows. In non-seizure episodes, the signal typical for epileptic discharges is missing at each electrode. This is a reason why MI values are approximately equal among all channels in the negative class (see Figure 7B). As an analogy, MI values slightly vary across different brain regions in generalized seizures in which bilateral EEG discharges appear symmetrically and synchronously over homologous regions of the head (see GNSZ in Figure 7G) [44].

In the studied sample, FNSZ episodes outnumbered other discharges. Although seizures can occur anywhere in the brain, the epileptic focal seizures generally occur at the seizure onset zone [44,45,46]. An ’epileptogenic zone’ is the region where epileptic seizures start or the part of the brain with the most ictal activity [45]. The epileptogenic zones of FNSZ are typically located in the temporal and frontal lobes, which explains high MI values in the correspondent areas (see Figure 7C).

The pathophysiology of absence seizures remains a matter of debate. According to the “centrencephalic” theory, these discharges originate from a deep-seated subcortical pacemaker in the midline thalamus. This theory was refined in 1991 with the “thalamic clock” theory, implying that the reticular thalamic nucleus contains pacemaker cells for the thalamic clock imposing its rhythm onto the cortex [47]. The theories can explain the projection of brain electric activity on the paramedial zones of the frontal, parietal and occipital lobes tightly connected to thalamus [48,49,50] (see MI topoplot for ABSZ in Figure 7H).

Partial seizures commonly originate from the temporal lobe [51]. We also observed abnormal electric activity in the temporal lobes in simple and complex partial seizures (see diagrams for SPSZ and CPSZ in Figure 7D,E). Sometimes, seizure semiology fails to identify the location of the abnormal electrical activity of the brain [44,51]. It is challenging to interpret AM patterns for subtypes of motor onset epileptic discharges. Only tonic seizures are diagnosed primarily from the abnormal EEG signal in the precentral and premotor cortex (see Figure 7I). Seizures arising from the motor system are organized in complex electrophysiological patterns that often involve the precentral cortex and lateral and mesial aspects of premotor areas [52].

Our network learned distinct patterns of electrophysiologic activity and placed different weights on electrodes according to the location of abnormal epileptic activity in seizure types. We looked for results that electrophysiologists could readily interpret. In the attempt to distinguish seizure types, we employed a source activation technique.

### 3.5. Brain Electrophysiologic Activity in Seizure Differentiation with Source Reconstruction

**Figure 8:**
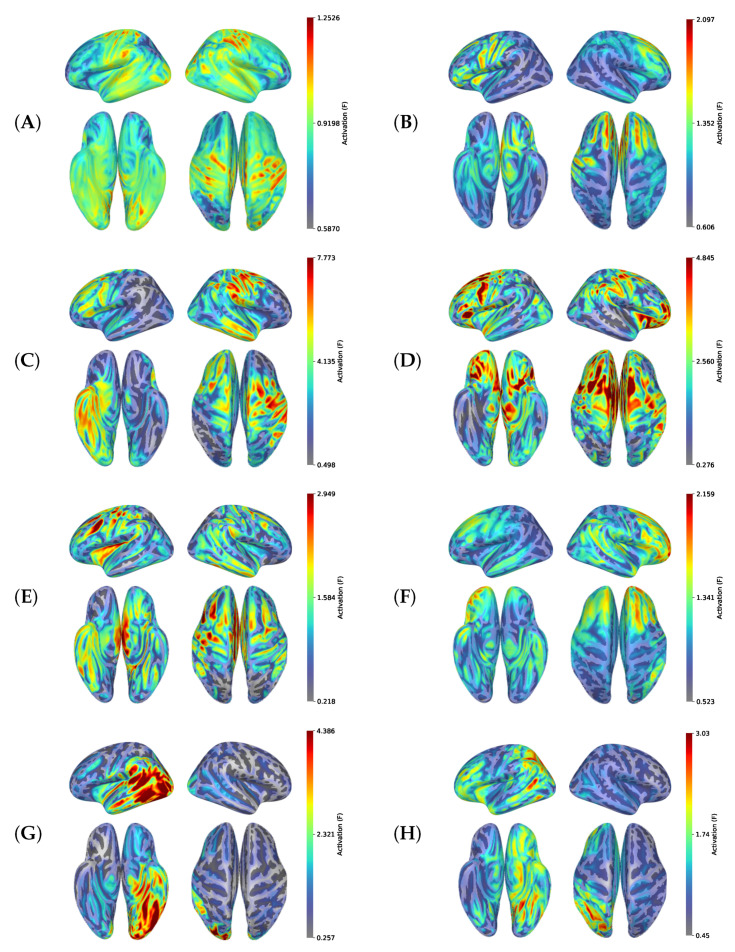
Activations of the sources averaged over the first two seconds of an epilepsy episode and reconstructed with DSPM method for different seizure types: GNSZ (**A**), ABSZ (**B**), MYSZ (**C**), TNSZ (**D**), TCSZ (**E**), FNSZ (**F**), SPSZ (**G**) and CPSZ (**H**).

With the source reconstruction technique, we illustrated the localization of brain areas with high neuronal activity within the first two seconds of seizure EEG recording (see Figure 8). *In generalized seizures (GNSZ, ABSZ, MYSZ, TNSZ, and TCSZ)*, source activity is spread over the whole cortex, and epileptic episodes originate from both brain hemispheres, but the location of the predominant activity differs among seizure types. Figure 8A shows a GNSZ with maximal source activity between the frontal and parietal cortex. Activity in the temporal cortex on both sides is slightly lower. In ABSZ, MYSZ, TCSZ and TNSZ depicted in Figure 8B–E, the marked activity is also located bilaterally in the cortex. *In the focal (syn. partial) seizures (FNSZ, SPSZ and CPSZ)*, epileptic activity starts from one of the hemispheres. Figure 8F–H shows an FNSZ case with dominant neuronal activity in the right frontal cortex, an SPSZ case with activity in the left occipito-temporal cortex, and a CPSZ case with activity in the cortex of the left hemisphere.

## 4. Discussion

### 4.1. Performance of Models for EEG-Based Seizure Detection and Classification

Scalp EEG is the most broadly used tool for diagnosing and monitoring epilepsy [53,54,55]. The disadvantages of scalp EEG are artifacts, signal noise, low spacial resolution and complicated analysis that can be improved with ML and DL models. The models can perform a quick and accurate automatic EEG assessment. However, the optimal model and the most advantageous EEG acquisition settings have not been described yet. In the current study, we proposed a system architecture for precise seizure detection and classification from raw EEG data of the TUSZ dataset. In comparison with the recent studies, we reported an increased classification accuracy. Another strength of our system architecture is that it establishes a straight-forward pipeline for two consecutive tasks: epileptic episode detection and classification. The latter is rarely reported in the available literature. Another advantage of the architecture of our study is that it allows us to apply interpretable ML.

We reported a highly sensitive detection of seizures (87.7%) with the binary (seizure/non-seizure) classification model. Non-seizure episodes were detected with higher sensitivity because of the unbalanced dataset. Still, the system missed only 12.3% of seizure episodes, which makes it a reliable tool that outperforms other models trained on the same dataset. For seizure detection, a study reported 79.10% Sn, 81.38% Sp and a true positive rate of 34.61% [56]. Another team documented a model with 72.59% Sn and 83.26% Sp [57]. Other authors showed a highly accurate model that discriminated GNSZ from non-seizure episodes (95.05–97.07% Sn, 94.91–94.56% Sp, and 94.98–95.79% Acc) [58]. Despite the high performance values, the clinical applicability of their model is questionable since it does not detect the other seven seizure types.

Recent papers on DL analysis of the TUSZ dataset commonly discuss findings on seizure detection but seldom present their classification by types. Some authors detected focal seizures in the TUSZ dataset with 23.02–37.61% Sn and generalized ones with 59.50–81.10% [57]. Our RNN distinguishes among eight seizure types with higher accuracy (average Acc of 96%) and outperforms their classification model.

As we know from clinical practice, EEG findings should be interpreted in combination with clinical data. Neurologists cannot distinguish SPSZ from FNSZ without clinical assessment. CPSZ is associated with impairment in consciousness, and these seizures can only be detected when clinical data are captured in the EEG report. The TUSZ dataset did not contain clinical findings, which explains the false detection of some seizure types. None the less, the system of automatic seizure detection can serve as a tool for computer-aided decisions made by physicians. The system can also report the episodes that remained unmentioned by the patient and medical staff.

For multigroup classification of epileptic seizures, we selected a model architecture compatible with interpretable ML. Similar solutions have been tested by different authors who reported discrepant results. Some researchers created an automated system based on discrete wavelet transform decomposition, Direct Quadrature and a neural-network-based ML model that uses features of Shannon Entropy. These methods proved highly accurate even in temporal and spectral disturbances. The researchers reported 100% Acc, Sn and Sp for the multiclass epilepsy classification from EEG signals (healthy, ictal, inter-ictal) [59,60]. Other scientists used a method based on variational mode decomposition (VMD) and a non-linear twin support vector machine (NLTWSVM) for epileptic seizure detection and classification [61]. The authors reported 99.2% Sn, 99.5% Sp and 99.4% Acc for differentiation between non-seizure and seizure EEG records of a publicly available online database from the Department of Epileptology, University of Bonn, Germany [62]. Another scientific team classified seven epileptic seizure types (FNSZ, GNSZ, SPSZ, CPSZ, ABSZ, TNSZ and TCSZ) with 89–98% Acc and a weighted score of 0.923 on the TUSZ dataset. Other researchers performed multiclass seizure classification (FNSZ, GNSZ, SPSZ, and TNSZ) of the TUSZ dataset with empirical mode decomposition (EMD) and a Support Vector Machine (SVM). They reached the highest Acc of 95% by using a quadratic SVM kernel [63]. In another study, a combination of graph neural networks (GNN) and meta-learning received 82.7% Acc and 0.82 F1 score for seizure detection and classification [64]. Other authors used adapted Meta Update Strategy (MUPS) and reached macro-F1 of 0.51 and AUC of 0.679 in four-class classification: non-seizure samples and three seizure types from the TUSZ dataset [65].

### 4.2. EEG Sampling Frequency in Seizure Detection and Classification

Systems for EEG analysis have been renovated over decades to detect seizures reliably. Authors have been searching for an optimal SR for EEG data collection [66,67,68]. Lower frequencies (173–200 Hz) are mainly used in clinical settings, while higher frequencies (10–32 kHz) are employed for research [69]. The high SR may enormously raise the complexity of ML models without an increase in performance. Our findings showed that performance did not improve significantly with an SR increase from 50 to 250 Hz. However, the top-performing ML algorithms were trained on the EEG data acquired at the SR of 250 Hz. The gain in precision was 5% in the binary classification model and 7% in the multigroup one (see Figure 4A,B, respectively). Still, a low SR can be used in fast diagnostic models with reasonably reliable performance. Different authors tested model performance at a low (64–128 Hz), moderate and high SR (up to 4 kHz) [70,71]. No association between the acquisition frequency and model accuracy was found. Other researchers established that the higher sampling frequency of scalp EEG also did not boost seizure detection. In their study, Acc, Sn and Sp differed by 1–2% for the SR values of 256, 512 and 1024 Hz [72]. In analogy to this, the rising acquisition frequency of the intracranial EEG improved neither detection of the seizure onset nor localization of epileptogenic foci [73]. Thus, an increase in SR is not accompanied by significant improvement in ML performance metrics.

### 4.3. Number of EEG Electrodes in Automatic Detection and Classification of Epileptic Episodes

The discussion about the number of scalp electrodes sufficient for reliable EEG recording is still open. Optimally, the selection of EEG electrodes should be individualized according to disease manifestation. Such an individual approach increases diagnostic sensitivity and minimizes artifacts and noise. Minimization of artifacts and noise reduction can be achieved with a decrease in the number of electrodes, although the lower EEG resolution can question its detective accuracy. This problem reveals a trade-off between spatial resolution and computational complexity. Some findings presented by different authors on the CHB-MIT dataset can be explained by the complexity of the models they chose. With 18 and 12 electrodes and five channels, they reported Sn 80.87, 95.70 and 97.91% respectively, and Sp 47.45, 96.55 and 99.57% [24,74,75]. Other researchers showed a stable model performance when the number of channels was increased from 6 to 10. Increasing the number of channels from 10 to 19, they gained a considerable model performance, i.e., the area under the curve rose from 91.57 to 98% [76].

A few references indicate a possibility to build accurate models with low spacial EEG resolution. For example, authors who analyzed the Rigshospitalet and Northzealand Hospital dataset with a three-electrode system reported brilliant performance: 98.40% Sn and 100% Sp [77]. A study of nocturnal frontal lobe epilepsy with 11 EEG channels also achieved reputable performance: 96.39% Acc, 93.20% Sn, and 96.81% Sp [78]. Models trained on six-channel EEG data from the Neurological Center Rosenhuegel showed 84.99–93.39% Sn and 90.05–95.60% Sp [79]. Other researchers wrote about 96.60% Sn and 92.50% Sp of seizure onset detection with only a two-EEG-channel system [80]. Some studies suggested using a six-channel system since it provides reliable detection of partial seizures (77.90% Sn) [81]. At the same time, several authors discussed worsening of performance with a reduced number of electrodes and channels. For example, analysis of the TUSZ dataset showed a gradual decline in Sn (from 39.15 to 31.15%) and Sp (from 90.37 to 40.82%) with 22, 20, 16, 8, 4 and 2 channels [16].

In our study, the metrics of the binary model remained high when the number of electrodes was decreased from 21 to 8. Diagnostic accuracy was stable due to common signal patterns detected by the electrodes, i.e., seizure detection was reliable with a moderately low number of EEG sensors. In contrast to this, the average performance of the multigroup classification model decreased significantly with reduced spatial resolution of EEG. Supposedly, focal seizures were not detected when the scalp was insufficiently covered with sensors. Our data are consistent with the literature findings: the top accurate system uses all channels from the 10–20 EEG configuration, and a reduction in the number of channels from 16 to 8 results in a small but measurable degradation in performance [16].

### 4.4. Activation Maximization for Seizure Detection and Classification

LSTM and EEGNet are the most common models for the detection of epileptic seizures. The models are often criticized for being black boxes because clinicians cannot use their outputs as references. A way to give precise seizure interpretation is to design convolutional layers and extract clinically relevant frequency bands from the characteristic filters. Initial seizure location is a significant feature for constructing interpretable DL algorithms [11]. The feature may contain discriminative information for systems to differentiate among seizure types; thus, it brings interpretability to an ML system [82]. We used similar features to explain the behavior of the system for automatic detection and classification of epileptic discharges. The MI values projected on topoplots justified the presence of EEG patterns that differ between seizure/non-seizure or among eight types of epileptic activity. Constructing the topoplots helps us to understand how the classification works and foster trust in the ML model. In particular, AM as a visualization technique increases model explainability by retrieving class-specific EEG features and presenting them graphically [83,84].

In Section 3.4, we suggested a pathophysiologic explanation for activation heatmaps. Visual ranking of low and hight level features throughout convolutional layers explains how the system works. However, a possibility to treat the decoded filters as diagnostic templates is questionable: AM maps may have a low spatial dependence on the signal localization and may depict a wholly non-localized area [85]. These limitations restrict the practical utility of AM frameworks: they only show how a DL model works.

The technique that we used for examining seizure-specific patterns was recently applied by other authors to electrophysiologic studies. However, they analyzed EEG data in another way: they attributed importance to different frequency bands and presented explainability approaches that provide an insight into the spectral features learned by DL classifiers [83]. Another study showed that AM can visualize a distinction in the theta frequency between sexes [86]. Other researchers reported that AM is also able to accurately differentiate among sleep stages [83] and between patients with schizophrenia and healthy individuals [87]. A study on brain–computer interfaces showed the advantages of implementation of AM for learning EEG patterns of the brain activities during four motor imagery tasks and a resting state [88]. Priyasad D. et al. used raw EEG signals and multi-channel attentive feature fusion to develop an interpretable DL architecture that classified epileptic seizures from the TUSZ. The authors reported a 0.966 F1 score, 96.65% Acc and 98.08% Sp for the multiclass seizure classification [89].

The potential of AM to reveal any bias in the model makes it an attractive tool for debugging an ML algorithm during the development phase. Despite the fact that the AM method is able to reflect the actual behavior of the model more intuitively, the resulting prototype samples can be difficult to interpret [82]. A straight-forward connection between the anatomo-physiological basis of epileptic discharges and model behavior is missing because of noise at the time of signal acquisition and uncertainty during the process of optimization [82,83]. Still, the extracted features may have a loose connection with anatomic and physiologic mechanisms underlying seizures.

### 4.5. Brain Electrophysiologic Activity in Seizure Differentiation with Source Reconstruction Technique

The source reconstruction technique enables us to differentiate between generalized and focal seizures. The latter can be treated with brain surgery [90,91,92]. The source reconstruction technique can localize an epileptogetic focus and guide the neurointervention. The intracranial EEG is the “golden standard” of seizure origin localization [93,94,95]. However, we performed EEG source imaging with scalp detectors that are more applicable in practice [94,96,97]. Scalp EEG can localize the initial epileptic focus with high accuracy, e.g., the LORETA algorithm [98], exact LORETA kurtosis [99,100,101], exact LORETA current source density analysis [101], and switching Markov model [102]. A study reported a highly accurate detection of epileptic foci with multielectrode scalp EEGs (128-256 channels). The described algorithm outperformed MRI, positron emission tomography and single photon emission computed tomography: 84 vs. 76 vs. 69 vs. 58% Sn and 88 vs. 53 vs. 44 vs. 47% Sp, respectively [97]. A weakness of the source reconstruction technique is that it may fail to differentiate FNSZ from TCSZ due to common epileptic activity. Another weakness is that artifacts from muscle movements during the seizure complicate the EEG source identification. Some seizure types can be classified only clinically by a physician. For example, the only difference between SPSZ and FNSZ is the state of consciousness [103].

A distinguishable feature of our research was the exceptional reconstruction of the EEG source during the seizure initiation. We wanted to remove from the analysis the time intervals when the epileptic activity spreads within the brain and recruits surrounding neurons. The propagation involves identical neural networks in different seizure types and complicates their classification with EEG [104]. Other researchers used spike peak analysis of scalp EEG data to localize an epileptic focus [98]. With this approach, they failed to receive interpretable images that could help to differentiate generalized seizures from the focal ones. New studies are required to test whether seizure onset analysis will improve classification into types.

### 4.6. Prospects of Interpretable Machine Learning for Medicine

The current challenges in the mass implementation of EEG appliances arise from disease heterogeneity among patients and technical limitations in long-term ambulatory monitoring. The existing frameworks were designed primarily for in-hospital use, and they require preprocessing of EEG data. The current wearable EEG monitors predominantly detect generalized tonic-clonic seizures, which is insufficient for proper patient management [105].

We see the following prospects for the implementation of interpretable ML. First, it can be embedded into clinical decision support systems. Second, the ML algorithms are suitable for creating wearable devices that will improve healthcare in outpatient clinics. Third, the algorithms can detect the pre-ictal period that does not have a generalized clinical manifestation. Reliable prognostication of such episodes will address the real patients’ needs and improve disease management. Fourth, identification of seizure types will enhance the existing AM-based CNN models that can accurately detect seizures with two channels, which makes them adaptable to a home-based seizure monitoring system [105,106,107].

## 5. Strengths and Limitations

The study strengths are as follows. First, our results provided insight into the optimal settings for acquiring the EEG signal. Second, we described advanced system architecture for precise epileptic seizure detection and classification. Third, we used interpretable ML to reach the aim. This helped us to explain the findings to clinicians, increase their trust in DL models and support future clinical implementation of the study findings.

We see the pros and cons of the non-overlapping window approach which we used. On the one hand, it ensured that training samples were analyzed separately from the test ones. Otherwise, some parts of the time windows on which the model was trained could be randomly included in the testing set during sampling. On the other hand, the non-overlapping approach could hide the temporal patterns at the borders between separate windows. Future studies should address the question of how the overlap between adjacent windows affects the model performance.

The current study had the following weaknesses. First, we analyzed an open-source dataset that did not contain MRI scans of the patients. For this reason, we had to use a standard template from a Python library to reconstruct EEG sources. The application of individual scans would be preferable. Still, our approach allowed us to reveal brain physiological activity. Second, the model architecture that we selected implied using non less than eight electrodes. Therefore, we could not test system performance with fewer sensors. Still, we managed to show a change in model quality with the reduction in the number of detectors. Third, we investigated model interpretability by decoding the filters learned along convolutional layers. This resulted in information loss due to feature extraction. We also could not consider individual variance in spectrum distribution of EEG data [106].

## 6. Conclusions

- In the available literature, we looked for models that optimally fit the following criteria: reliable performance, ability to generalize on a broad set of EEG recordings, quick processing of the input data, high explainability and interpretability. We selected the top-performing models to construct a system for automatic seizure detection and classification.
  The system accurately detects seizure episodes (87.7% Sn, 91.16% Sp) and carefully distinguishes eight seizure types (95–100% Acc).
- An increase in EEG sampling rate from 50 to 250 Hz boosted model performance: the precision of seizure detection rose by 5% and seizure differentiation by 7%. A low sampling rate is a reasonable solution for training reliable models with EEG data.
- Decreasing the number of EEG electrodes from 21 to 8 did not affect seizure detection but significantly worsened seizure differentiation. Different electrodes were equally informative in detecting epileptic episodes but not as effective in differentiating among seizure types. However, the optimal number and location of sensors depends on individual clinical signs.
- We improved model explainability with interpretable ML: topoplots displaying neuron maximization showed evident differences between seizure and non-seizure classes. Activation maximization highlighted the presence of EEG patterns specific to eight seizure types. With interpretable ML, we justified that our system recognizes biologically meaningful features as indicators of epileptic activity in EEG.
- The source reconstruction technique further improved the model explainability by supplying us with the cortical projection of epileptic sources in the brain. The received cortical projections depicted EEG source differences between the onset activity in generalized and focal seizures. New studies are required to test whether seizure onset analysis will improve classification into types.

## Figures and Tables

**Table 1:** Performance of machine learning algorithms for seizure detection.

Reference	Year	Database	Method	Sampling Rate, Hz	Metrics		
					Accuracy, %	Sensitivity, %	Specificity, %
**Conventional Machine Learning**							
[24]	2019	CHB-MIT	SVM Naive Bayes	256	95.63	95.7	96.55
[25]	2017	UoB	DT-CWT	173.6	98.87	98.20	100
[26]	2012	UoB	FSC	173.6	98.1	99.4	100
			SVM		95.9	97.2	100
			kNN		93	97.8	97.8
			PNN		93	97.8	97.8
			DT		88.5	98.3	91.1
			GMM		95.9	98.3	95.6
			DBC		97.8	94.4	97.8
[27]	2018	XJU	WDTF	256	99.4	92.1	99.5
[28]	2018	UoB	HVD	173.6	97.66	98	98
[29]	2015	MCH	GLM	200 500 512	87.5	88.8	85.7
[30]	2015	UoB	MLPNN SVM kNN+NLFV	173.6	96.5 94.8 98.4	96.5 96.5 99	96.5 93 97.9
[31]	2019	UoB	LS-SVM	173.6	99.5	100	99.4
[32]	2018	TWH	CFC Morlet	500 1024	82.4	87.9	82.4
[33]	2015	CHB-MIT	LDA	256	94.69	89.1	94.8
**Deep Learning**							
[34]	2019	CHB-MIT	2D CNN	256	98.05	90	91.65
[35]	2019	UoB	DNN	173.6	97.21	98.59	91.47
[36]	2020	UoB	SEA-based DNN	173.6	97.17	93.11	98.18
[37]	2021	TUSZ CHB-MIT UoB	EEGWaveNet	256 256 173.6	67.68 96.17 99.89	59.21 65.83 99.80	75.30 96.96 99.97
[38]	2019	UoB	ESD-LSTM	173.6	100	100	100
[39]	2018	UoB	P-1D-CNN	173.6	99.1	96	98
[40]	2019	XMUH	LRCN	500	93.4	91.88	86.13
[41]	2019	CHB-MIT	2D CNN 3D CNN	256	98.33	96.66	99.14
[42]	2019	CC	TGCN	200	98.05	90	91.65
[43]	2020	FED	BILSTM	256	98.69	98.09	98.69

Abbreviations: MCH—Miami Children’s Hospital; CHB-MIT—Children’s Hospital Boston and the Massachusetts Institute of Technology; UoB—University of Bonn; XJU—Xian Jiaotong University; TUSZ—Temple University Hospital EEG Corpus; TWH—Toronto Western Hospital; TWH—Toronto Western Hospital; XMUH—Xinjiang Medical University Hospital, CC—Clevelend Clinic; FED—Freiburg Epilepsy dataset.

**Table 2:** Electrodes ranking according to MI significance scores.

Binary Classification		Multiclass Classification	
**Electrode**	**MI Significance Score**	**Electrode**	**MI Significance Score**
C4	0.006894	Fz	0.178724
F4	0.006632	Pz	0.178065
T3	0.006631	Fp2	0.177629
Fp2	0.006592	T4	0.176386
P4	0.006588	O2	0.176262
F3	0.006522	C4	0.176049
O1	0.006444	F4	0.175952
A1	0.006419	F8	0.175667
C3	0.006405	F3	0.175562
T6	0.006347	T5	0.175543
T5	0.006268	O1	0.175284
P3	0.006251	A2	0.175005
T4	0.006234	Cz	0.174934
Fp1	0.006216	P4	0.174509
O2	0.006126	P3	0.174291
A2	0.006085	A1	0.174280
Cz	0.006038	F7	0.173992
F8	0.006025	T3	0.173690
Pz	0.006023	C3	0.173577
Fz	0.005865	T6	0.173267
F7	0.005654	Fp1	0.172294

## Data Availability

The datasets and code generated for this study are available on request at the site of Big Data Analytics Center (BIDAC) at https://bi-dac.com (accessed on 20 July 2023).

## Supplementary Materials

The following supporting information can be downloaded at: https://www.mdpi.com/article/10.3390/biomedicines11092370/s1, Figure S1: Number of electrodes in EEG recordings from TUSZ dataset; Table S1: Performance of CNN binary classification model at different sampling rates [108,109,110,111].

## Abbreviations

The following abbreviations are used in this manuscript:

ABSZ	absence seizure
Acc	accuracy
AM	activation maximization
ATSZ	atonic seizure
CNN	convolutional neural network
CNSZ	clonic seizure
CPSZ	complex partial seizure
DL	deep learning
EEG	electroencephalography
FNSZ	focal non-specific seizure
GNSZ	generalized non-specific seizure
LSTM	long short-term memory
MI	mutual information
ML	machine learning
MRI	magnetic resonance imaging
MYSZ	myoclonic seizure
NESZ	non-epileptic seizure
RNN	recurrent neural network
Sn	sensitivity
Sp	specificity
SPSZ	simple partial seizure
SR	sampling rate
STFT	short time Fourier transform
TCSZ	tonic-clonic seizure
TNSZ	tonic seizure
TUSZ	Temple University Hospital EEG Seizure Corpus
