# Supplementary material for: Automatic Detection and Classification of Epileptic Seizures from EEG Data: Finding Optimal Acquisition Settings and Testing Interpretable Machine Learning Approach
Source: Biomedicines. 2023 Aug 24;11(9):2370. doi: 10.3390/biomedicines11092370 (PMC10525492; doi:10.3390/biomedicines11092370)
Supplement: Supplementary file 1 [file biomedicines-11-02370-s001.zip › SupplementaryMaterials.pdf]

Supplementary Materials:

1. Details on Study Methodology

1.1. Data Description and Formulation of Problem in Terms of Input and Output

EEG gave rise to a rich set of data from the individual discharges detected with electrodes across the entire span of the scalp. Computational analysis was performed with two data types: european data format (EDF) and comma-separated values (CSV). EDF stores time series of EEG signals received from long-term monitoring (LTM standard). Information stored in EDF helped to diagnose epilepsy by correlating the time of physical manifestation of seizures with abnormal electrical activity in the brain. Each channel was called "EDF Annotations" and it corresponded to a certain electrode parsed and extracted into the RAW image format for further processing. A CSV file imported into a python script contained data on seizure types, start/stop timing and electrodes. Figure S1 illustrates the number of electrodes in different montages used for data collection in the TUSZ dataset. We decided to work with 21 electrodes of the international standard 10/20 system. The decision allowed us to analyze recordings of all the patients except for two individuals who were examined with a 20-electrode system. For setting the 10/20 montage we used functions from MNE package.

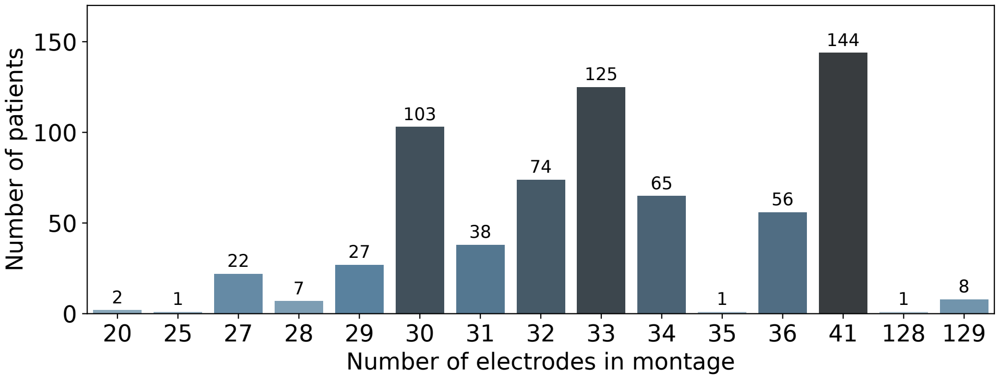

Figure S1. Number of electrodes in EEG recordings from TUSZ dataset

For preprocessing the data for binary and multigroup models, we used the 'non-overlapping window' technique. Specifically, we slided a window of size 250 over a time series and extracted 250×21 matrices, where $f = 21$ was the number of features and $n = 250$ was the number of timepoints within a one-second-long window at the sampling rate of $F_s = 250$ Hz. Then, each sliding window was processed with a 64-point short time Fourier transform (STFT) and the power density spectrum was stored into a 3D tensor of size $250 \times 21 \times 33 \times 8$ where 250 was the window length, 21, 33 and 8 was the number of channels, frequencies and timepoints respectively.

1.2. Methods Used

We used two main architectures For constructing deep learning (DL) models: a convolutional neural network (CNN) for the binary classification and a recurrent neural network (RNN) for the multigroup classification. CNN architecture is able to learn highly abstract features of objects, which makes it appropriate for our task since EEG data can be converted into the RAW format for computer vision analysis and patterns recognition. The model we built mainly consisted of three blocks each of which had a convolution layer and a max pooling layer. If compared to a big number of pretrained models developed for DL in Python, our solution provided higher flexibility for dealing with a non-standard input

image size. The convolutions used a three-dimensional filter. In the binary classification, the filter slided over time series of EEG and computed a dot product between the pixels and the weights detecting seizure and non-seizure episodes. An advantage of using a three-block CNN for computer vision was the network ability to learn features from raw images at different levels of abstraction. The first block learned simple features (edges), while the second one learned more complex features (shapes). The final fully-connected layer classified images based on the extracted features. The architecture reduced a need for hyperparameters, hand-engineered filters, and matrix operations speeded up the evolutionary process dramatically keeping performance metrics high.

The multigroup classification required a more sophisticated model. We decided to use the long short-term memory (LSTM) architecture. Input EEG data were stored as a tensor which was a three-dimensional array of the following matrices: channels, patients and timestamps. We used Tensorflow framework to build models and make predictions from the data structured in tensors. When the input gap is large, sigma or tanh cells of the standard RNN are unable to learn the relevant information of input data. In contrast to this, LSTM handles the problem of long-term dependencies well. Stacking multiple cells improves the performance on sequences such as EEG time series with different frequencies. The model has a bidirectional internal mechanism that processes the input data forward and backward. In this way, the model can access past and future context to improve prediction.

From Tensorflow, we took the following key functions for building ML model. First, *BatchNormalization* is a function which transforms input data maintaining mean output close to 0 and output standard deviation close to 1. Second, *ConvLSTM2D* is a layer that combines the LSTM gating with 2D convolutions, performs convolution operations and retains input dimensions. In our study, dimensionality of output space (number of filters) was set to 128 while the dimension (kernel size) of the convolution window was (5, 1). Third, *MaxPooling3D* is a tool for downsampling the input along its spatial dimensions (depth, height, and width) by taking the maximum value over an input window for each input channel. Fourth, *a Dropout layer* is a set of functions used to avoid overfitting. At each training step, the layer randomly sets input units to 0. The system calculates a rate of dropouts applied to random neurons. The inputs not set to 0 are scaled up by 1/(1 - rate) so that the sum over all inputs remains unchanged.

After the block of functions described above, we implemented a similar block with other attribute values. These two blocks differed in the number of filters in ConvLSTM2D: it was equal to 64 in the second LSTM cell. After that, we put *a Dense layer* which performed element-wise activation. Finally, we incorporated *a Flatten layer* that reduced the input data into a single dimension and *an Activation layer* which applied Softmax activation function for the multiclass classification.

We tested model performance with different acquisition frequency settings, sliding windows and strides. A downsampling function decreased the sample rate by keeping the first time window and then every n'th sample following it. A sliding window indicated the position where a particular time series interval had to be displaced. This position was controlled by a coefficient relative to the total number of windows, whereas a shift inside the sliding window was controlled an index of the record. These variables defined a window length and a displacement parameters. We trained the models and saved the results in .h5 files.

1.3. Evaluation Metrics

Mutual Information (MI) for feature significance analysis. In feature significance analysis, we used the mutual information (MI) metric. MI can capture any kind of dependencies between variables and targets, including nonlinear relationships and it is invariant to data transformations [1]. MI reflects how much the value of one variable reduces the uncertainty on the other [2], which gives an idea about relevance of a feature subset to targets. The MI score is defined as below:

$$I(x,y) = \sum_{i=1}^{n} \sum_{j=1}^{n} p(x_i, y_j) \cdot \left(\frac{p(x_i, y_j)}{p(x_i)p(x_j)} \right), \tag{1}$$

where $p(x_i)$ is a mass probability of a discrete random variable x: $p(x_i) = \Pr\{x = x_i\}, x_i \in x$ [1].

The MI is 0 when the random variables are statistically independent. The mutual information between a set of m features and the class variable Y is computed as:

$$I(\{x_1, \dots, x_m\}, Y) = \sum_{k=1}^{m} \sum_{\substack{S \subseteq \{x_1, \dots, x_m\}, \\ |S|=k}} I([S \cup Y]), \tag{2}$$

where $I([S \cup Y]) = I(s_1, s_2, \dots s_k, Y)$.

Performance evaluation metrics. The model performance was evaluated with specificity (TNR - true negative rate), sensitivity (TPR - true positive rate), F1-score and confusion matrices. The first three metrics are relative scores. The higher the scores are, the better the model distinguishes between classes. An excellent model has a score near to 1 which means a good measure of separability. In multiclass classification, a notion of TNR or TPR is obtained only after binarizing the output in any of two possible ways. The 'One-vs-Rest' scheme compares each class against all others assumed as one, and the 'One-vs-One' scheme compares each unique pairwise combination of classes.

We used several objective measures to evaluate the performance of classification methods. A confusion or error matrix was built for each predictive model to show how it can distinguish classes. ROC AUC were used to evaluate classifier performance and summarize the trade-off between the true positive rate (TPR) and false positive rate (FPR) with different probability thresholds. The medical decision making community has an extensive literature on ROC graphs for diagnostic testing of balanced data [3,4]. For this reason we also found this metric appropriate for our study. In the binary data matrix, Cij is equal to the number of observations known to be in group "i" and predicted to be in group "j". The count of true negatives is C00, false negatives — C10, true positives — C11 and false positives — C01. A multiclass matrix is designed in a similar way however the data are treated as if they are binarized under the 'One-vs-Rest' transformation.

Performance metrics were calculated as below:

$$TPR(sensitivity) = \frac{TP}{TP + FN} \tag{3}$$

$$TNR(specificity) = \frac{TN}{TN + FP} \tag{4}$$

$$FPR(false\ positive\ rate) = \frac{FP}{FP + TN} = 1 - specificity \tag{5}$$

$$BAC(balanced\ accuracy) = \frac{Sensitivity + Specificity}{2} \tag{6}$$

The overall accuracy of the model was defined as follows:

$$Accuracy = \frac{TP + TN}{TP + TN + FP + FN} \tag{7}$$

where TP, TN, FP, FN are true positive, true negative, false positive and false negative values representing the confusion matrix of classification model respectively.

In each fold, 20% of training data were used for validation and the remaining 80% were used as the testing dataset. All metrics were calculated for each fold separately and then averaged values served as final metrics.

2. Results

2.1. Impact of EEG Sampling Frequency on Seizure Detection and Classification

Table S1. Performance of CNN binary classification model at different sampling rates

Metrics	Sampling rate, Hz				
	50	100	150	200	250
**Binary classification model**					
Sensitivity	0.8696	0.8585	0.8647	0.8700	0.8768
Specificity	0.8830	0.8943	0.9131	0.9103	0.9149
Precision	0.8168	0.8297	0.8565	0.8535	0.8605
F1 score	0.8423	0.8438	0.8605	0.8616	0.8685
**Multigroup classification model**					
Accuracy	0.8876	0.9259	0.8708	0.9325	0.9580
Precision	0.8664	0.8714	0.8548	0.8909	0.9371
Recall	0.9469	0.9122	0.9372	0.9550	0.9824
F1 score	0.9019	0.8787	0.8837	0.9178	0.9580

Abbreviations

The following abbreviations are used in the Supplementary Materials:

Acc	accuracy
AM	activation maximization
AUC	area under the ROC curve
BAC	balanced accuracy
CSV	comma-separated values
CNN	convolutional neural network
DL	deep learning
EDF	european data format
FN	false negatives
FP	false positives
FPR	false positive rate
LSTM	long short-term memory
LTM	long-term monitoring
MI	mutual information
RNN	recurrent neural network
ROC	receiver operating characteristic
STFT	short time Fourier transform
TN	true negatives
TNR	true negative rate
TP	true positives
TPR	true positive rate
TUSZ	Temple University Hospital EEG Seizure Corpus

1. Vergara, J.; Estevez, P. A Review of Feature Selection Methods Based on Mutual Information. *Neural Computing and Applications* **2014**, *24*. https://doi.org/10.1007/s00521-013-1368-0.
2. Beraha, M.; Metelli, A.M.; Papini, M.; Tirinzoni, A.; Restelli, M. Feature Selection via Mutual Information: New Theoretical Insights. In Proceedings of the 2019 International Joint Conference on Neural Networks (IJCNN), 2019, pp. 1–9. https://doi.org/10.1109/IJCNN.2019.8852410.
3. Fawcett, T. ROC graphs: Notes and practical considerations for researchers. *Machine learning* **2004**, *31*, 1–38.
4. Saito, T.; Rehmsmeier, M. The precision-recall plot is more informative than the ROC plot when evaluating binary classifiers on imbalanced datasets. *PloS one* **2015**, *10*, e0118432.
